# Mapping Late Leaf Spot Resistance in Peanut (*Arachis hypogaea*) Using QTL-seq Reveals Markers for Marker-Assisted Selection

**DOI:** 10.3389/fpls.2018.00083

**Published:** 2018-02-05

**Authors:** Josh Clevenger, Ye Chu, Carolina Chavarro, Stephanie Botton, Albert Culbreath, Thomas G. Isleib, C. C. Holbrook, Peggy Ozias-Akins

**Affiliations:** ^1^Center for Applied Genetic Technologies, Institute of Plant Breeding, Genetics and Genomics, University of Georgia, Athens, GA, United States; ^2^Department of Horticulture, Institute of Plant Breeding, Genetics and Genomics, University of Georgia, Tifton, GA, United States; ^3^Department of Plant Pathology, University of Georgia, Tifton, GA, United States; ^4^Department of Crop and Soil Sciences, North Carolina State University, Raleigh, NC, United States; ^5^United States Department of Agriculture-Agricultural Research Service, Tifton, GA, United States

**Keywords:** *Arachis*, QTL-seq, late leaf spot, polyploidy, resistance

## Abstract

Late leaf spot (LLS; *Cercosporidium personatum*) is a major fungal disease of cultivated peanut (*Arachis hypogaea*). A recombinant inbred line population segregating for quantitative field resistance was used to identify quantitative trait loci (QTL) using QTL-seq. High rates of false positive SNP calls using established methods in this allotetraploid crop obscured significant QTLs. To resolve this problem, robust parental SNPs were first identified using polyploid-specific SNP identification pipelines, leading to discovery of significant QTLs for LLS resistance. These QTLs were confirmed over 4 years of field data. Selection with markers linked to these QTLs resulted in a significant increase in resistance, showing that these markers can be immediately applied in breeding programs. This study demonstrates that QTL-seq can be used to rapidly identify QTLs controlling highly quantitative traits in polyploid crops with complex genomes. Markers identified can then be deployed in breeding programs, increasing the efficiency of selection using molecular tools.

**Key Message:** Field resistance to late leaf spot is a quantitative trait controlled by many QTLs. Using polyploid-specific methods, QTL-seq is faster and more cost effective than QTL mapping.

## Introduction

Peanut (*Arachis hypogaea*) is an important oil crop rich in protein, vitamins and minerals. It is grown in most temperate to sub-tropical regions of the world reaching a total of 40 million tons in global production in 2014^1^. Late leaf spot [LLS; caused by *Cercosporidium personatum* (Berk. & Curt.) Deighton] is a major fungal disease plaguing peanut production worldwide. In Georgia-United States, the disease starts around mid-August when the fungal spores germinate and penetrate peanut leaves through stomata located on the abaxial surface. Round-shaped black lesions become visible within 1 week of fungal germination. Since the spores are deposited in the soil, LLS disease usually starts from the bottom layer of the plant canopy and moves upward. As the fungal lesions enlarge, coalescence of lesions often is observed in highly susceptible lines (Gill, 2013). Sporulation occurs 20 to 30 days after infection. Secondary infection from freshly produced spores is common in the long peanut growing season. Reduction in photosynthesis due to fungal growth and subsequent defoliation decreases peanut production. Highly susceptible lines can lose all their leaves one month prior to maturity. It is estimated that LLS causes 30–70% yield penalty without spraying susceptible cultivars with fungicide (Singh et al., 2011). To control the disease, multiple fungicide sprays are needed throughout the growing season which incurs the highest cost for peanut disease management (Woodward et al., 2014) in addition to environmental pollution. Therefore, integrating host resistance into elite peanut cultivars would be the most effective solution to this fungal disease.

Breeding peanut for disease resistance is challenging since peanut germplasm has narrow genetic diversity (Pandey et al., 2012). Strong resistance to a wide range of diseases is harbored by wild diploid relatives of peanut; however, introgression of disease resistance alleles from wild diploids to allotetraploid peanut is hampered by the barrier of ploidy level differences (Proite et al., 2007). In spite of this limitation, there are a few examples of successful introgression of strong host resistance to diseases into cultivated peanut from wild diploid relatives (Stalker et al., 2013). As an example, root-knot nematode resistance in peanut is conferred by a large alien introgression on chromosome A09 from *A. cardenasii*, a wild A-genome diploid relative to cultivated peanut (Simpson et al., 1993; Nagy et al., 2010). In terms of host plant resistance to LLS, cultivated peanut germplasm PI203396 provided resistance alleles to LLS and led to the release of resistant cultivar Georganic (Holbrook and Culbreath, 2008). Meanwhile, LLS resistance in wild diploids such as *A. cardenasii* (Company et al., 1982), *A. diogoi* (Kumar and Kirti, 2015), *A. batizocoi* (Zhou et al., 2016), and *A. stenosperma* (Leal-Bertioli et al., 2009) has been identified. To access this resistance, induced allotetraploids were established by interspecific hybridization and used in breeding programs (Company et al., 1982; Stalker and Beute, 1993; Gowda et al., 2002; Tallury et al., 2014; Zhou et al., 2016). The most effective and widely deployed LLS resistance was introgressed from *A. cardenasii* initially chosen for early leaf spot resistance (Company et al., 1982). Leaf spot resistant line CS16 from this interspecific hybrid source was distributed to India (Stalker, 2017) and selected for LLS resistance resulting in two ICRISAT germplasm lines ICGV 86855 (CS16) and ICGV 86687 (CS 16 – B2 – B2) (ICRISAT Annual Report, 1986). CS16 was the progenitor of an Indian germplasm line, GPBD 4 (Gowda et al., 2002). Population TG24 x GPBD 4 was mapped for LLS resistance by SSR markers and QTL-seq (Khedikar et al., 2010; Sujay et al., 2012; Pandey et al., 2016), and LLS resistance QTLs explaining 40–60% of phenotypic variation were identified. The major QTL located at 131 to 135 Mbp of chromosome A03 was confirmed by QTL-seq (Pandey et al., 2016). Direct evidence of *A. cardenasii* origin for this QTL was provided by comparing IAC322, another CS16-derived line, and *A. cardenasii* using diagnostic SNPs identified with the IntroMap pipeline as well as SNP markers from the *Arachis* 58K SNP array (Clevenger et al., 2016, 2017). This genetic evidence further substantiated the origin of this reliable LLS resistance source as being created in the United States and preserved and selected further in India. Another disease resistant germplasm, GP-NC WS 16, was selected for its excellent early leaf spot resistance from these *A. cardenasii* introgressed lines (Tallury et al., 2014). It was one of the eight unique male parents paired with either Tifrunner (Holbrook and Culbreath, 2007) or Florida-07 (Gorbet and Tillman, 2009) to establish recombinant inbred populations as genetic mapping resources (Holbrook et al., 2013). Florida-07 is susceptible to LLS and the population C1801 = Florida-07 x GP-NC WS 16, segregating for disease response, was used to study genetic control of LLS resistance in this research project.

Bulk segregant analysis, the method of using bulked individuals that share a phenotype to identify markers tightly linked to a trait of interest was first pioneered in lettuce and tomato using amplified fragment length polymorphism (AFLP) and restriction fragment length polymorphism (RFLP) markers (Giovannoni et al., 1991; Michelmore et al., 1991). This method was combined with next generation sequencing (NGS) to map mutations to functional variant resolution using SHOREmap (Schneeberger et al., 2009). The SHOREmap methodology was then extended as mapping-by-sequencing to map functional variants in non-model organisms (Galvão et al., 2012). Finally, QTL-seq was proposed as a fast method to identify and fine map QTL (Takagi et al., 2013). The resolution of QTL-seq relies on the amount of recombination captured within the population and the number of individuals available to be bulked. The size of the bulks are a direct result of the quantitative nature of the trait of interest combined with resolution of phenotyping.

QTL-seq has been a successful tool to identify QTLs in diploid crop species (Lu et al., 2014; Illa-Berenguer et al., 2015; Singh et al., 2016). The duplicated nature of allopolyploid genomes can cause problems with false-positive SNP calls masking significant QTLs. Recently, QTL-seq was utilized to map leaf spot and rust resistance in peanut (Pandey et al., 2016). The region identified co-localizes with an alien introgression from the diploid A-genome species, *A. cardenasii* (Clevenger et al., 2017) that was introduced through the parent GPBD 4, and so the identification of this major QTL is less complex. Identification of alien introgressions from interspecific populations is straightforward compared to identifying polymorphisms between cultivated peanut chromosomes. This is because of the divergence of A or B genome wild progenitors is much greater than between cultivated genomes. Pandey et al. (2016) identified an alien introgression that contributed resistance to rust and leaf spot, but not a QTL originating from cultivated germplasm. As such the problem of false positive SNP calls within the introgressed region is mollified. Identification of minor QTL from *A. hypogaea* × *A. hypogaea* crosses has not previously been reported using QTL-seq. Identification of these QTL would be beneficial to breeding programs as a fast and efficient method of identifying markers strongly linked to traits of interest for use in marker-assisted breeding programs.

In this study, QTL-seq was used to identify tightly linked markers to LLS resistance in peanut. Methods of SNP identification that work well for diploid species produced too much false positive noise to identify significant QTLs. However, use of a polyploid-specific pipeline to identify markers between parental genotypes allowed sufficient resolution to identify three QTLs. These QTL were confirmed using marker-assisted selection within one-half of the recombinant inbred line (RIL) population not used for QTL-seq and by backwards selection. Four years of field data showed that these markers are suitable for marker-assisted selection.

## Materials and Methods

### Plant Materials and LLS Phenotyping

The C1801 (Florida-07 x GP-NC WS 16) population was advanced using small plots of bulked seed to minimize attrition (Holbrook et al., 2013). A random individual plant was harvested from each F_6_ to provide seed for line increases. F_6:8_ RILs were used to initiate phenotyping. At the F_2_ stage, the population was divided so that half of the population was advanced in Tifton, GA, United States and the other half was advanced in Raleigh, NC, United States. Subsequently, a total of 192 and 191 RILs were independently increased at these two locations.

Field phenotyping for LLS severity was performed with the 192 RILs advanced in Georgia according to a randomized block design with three field replications. The RILs were planted in twin-row plots (1.5 m × 1.8 m = 2.7 m^2^) at a seeding rate of six seeds per 0.3 m in Georgia in 2012, 2013, 2014, and 2015. Tests for years 2012 and 2013 were conducted at the University of Georgia College of Agricultural and Environmental Sciences Gibbs Farm, Tifton, GA, United States and for years 2014 and 2015 at the CAES Bowen Farm, Tifton, GA, United States. Marker-selected NC advanced lines along with resistant checks Georganic (Holbrook and Culbreath, 2008) IAC322, and GP-NC WS 16 and susceptible checks Florida-07 and IAC886 were tested at the Gibbs Farm, Tifton, GA, United States in summer 2016. No fungicide was applied during the growing season and LLS disease progression was evaluated according to the Florida 1 to 10 scale (Chiteka et al., 1988). Disease ratings started once LLS disease symptoms were identified in the population. Four ratings at an interval of 10 to 14 days were taken each year. Area under the disease progression curve (AUDPC) was calculated for each line. Year 2013 data was excluded from genetic mapping due to insufficient disease pressure.

### Statistical Analysis of Phenotype Data

Univariate variance analyses with GLM method was performed and the variance components were determined by restricted maximum likelihood (REML). The broad sense heritability was estimated according to the formula: *H*^2^ = σ_g_^2^/(σ_g_^2^ + σ^2^_gxe_/n+ σ^2^_e_/nr), where σ_g_^2^ was the genetic variance component among the RILs, σ^2^_gxe_ was the RIL x environment interaction variance component and σ^2^_e_ was the residual component, *n* was the number of environments and *r* was the number of replications (Hallauer and Miranda, 1988). Statistical analysis of phenotypic data was performed with SAS software version 9.4 (SAS Institute Inc., Cary, NC, United States). Normality of data distribution was tested by the Shapiro test.

### Re-sequencing

DNA was extracted from leaves of each individual to be bulked using Qiagen DNAeasy Plant mini kit^®^. Equal amounts of DNA were pooled to form each bulk. Whole genome shotgun sequencing libraries were constructed using the Illumina TruSeq PCR-free kit starting with 2 μg of total DNA isolated from single plants using Qiagen DNAeasy Plant mini kit^®^ and and paired-end 150 sequencing was performed on the Illumina HiSeq 2500 V4^®^ sequencer at HudsonAlpha Institute for Biotechnology (Huntsville, AL, United States). Florida-07 and GP-NC WS 16 had been integrated in a group of 20 genotypes for whole genome re-sequencing, including 10 parents of RIL populations (Holbrook et al., 2013) and 10 other genotypes with different traits of interest for breeding purposes (Clevenger et al., 2016).

The raw sequences were filtered and trimmed using Cutadapt v1.2.1. for adaptor trimming and TrimGalore v0.3.7. for quality trimming. About 88% of high quality reads mapped over the two diploid genomes (A and B genomes represented by *A. duranensis* and *A. ipaensis*^2^) with Bowtie2 using default parameters for sensitive local alignment reporting best alignment and zero mismatch in the 20 bp seed. Consequently, very similar overall alignment rate was obtained for both genomes, being 96.7% on average over the *A. duranensis* genome and 96.9% over the *A. ipaensis* genome. The SNP calling between all the genotypes based on the reference genomes, and filtering of homeologous SNPs was developed following the SWEEP Prime version program (Clevenger and Ozias-Akins, 2015), which includes Samtools v0.1.9 and Bcftools v0.1.9, using default parameters with – *ultimate* option and minimum depth of 5x.

A total of 98,966,134 2 × 125 paired-end reads for the resistant bulk and 140,618,299 paired-end reads for the susceptible bulk were mapped to a concatenated *in silico* synthetic tetraploid genome comprised of the *A. duranensis* and *A. ipaensis* pseudomolecules (Bertioli et al., 2016^2^) using Bowtie 2 v. 2.2.3 (Langmead and Salzberg, 2012) and default parameters. All SNPs were called using Samtools mpileup (Li et al., 2009).

The sequence from both bulks are available at The National Center for Biotechnology Information (NCBI) under the BioProject ID PRJNA419937 and can be accessed at the link http://www.ncbi.nlm.nih.gov/bioproject/419937.

### Analysis without Parental SNPs

An initial analysis was done without knowledge of parental SNPs. First, the resistant and susceptible bulk SNPs were filtered for ‘polymorphic’ loci using mpileup-generated genotypic probabilities using custom scripts and filtered for at least 10 reads per bulk covering a SNP. The SNP Index was calculated for each bulk by counting the number of reads with the SNP and dividing by the total reads mapped to the locus for each bulk. Then the susceptible bulk index was subtracted from the resistant bulk index to get the ΔSNP for each SNP.

### Analysis Using Parental SNPs and SWEEP

To clean up noise in the data from false-positive SNP calls, a new strategy was employed using parental SNPs identified with the polyploid-specific SNP filtering tool, SWEEP (Clevenger and Ozias-Akins, 2015). SNPs were identified between Florida-07 and GP-NC WS 16 using the re-sequencing data and SWEEP with the following parameters: ‘-s 1 -d 5 -r 0 –ultimate’. Using custom scripts the GP-NC WS 16 allele was designated the ‘resistant’ allele and the Florida-07 allele the ‘susceptible’ allele. The SNP indexes were recalculated for the two bulks using only the SNPs in common between the parental SNPs and the SNPs present in the bulks. A smoothing function to reduce noise in the data was carried out by using a sliding window average across each chromosome of ΔSNP with a window size of 2 Mb and interval of 500 kb (Supplementary File S2).

### Permutation Test for Significance

To generate a null model assuming no QTLs, a permutation test was carried out as in Takagi et al. (2013). Briefly, for each marker, 1,000 simulations were carried out by sampling alleles from a population of 200 RIL lines for two bulks by sampling a binomial distribution assuming 1:1 marker segregation. Then the alleles sampled from the bulks were simulated at the given depth at the marker of interest and ΔSNP was calculated. Two separate simulations were carried out to generate marker-specific thresholds for *p* < 0.05 and *p* < 0.01. The simulation python script is available in Supplementary File S1.

### Calculation of the G Statistic

The G statistic was calculated for each SNP as described in Magwene et al. (2011) using a custom python script. The script used is available in Supplementary File S1.

### Bootstrapping Simulation

The LLS rating data from all 4 years were shuffled 10,000 times and the top 16 individuals were selected to represent the ‘resistant’ lines and the bottom sixteen individuals were selected to represent the ‘susceptible’ lines. Sixteen was used because that is the number of individuals represented in the RIL population with resistant or susceptible alleles at each marker. For each iteration, the number of groups of shuffled individuals that had an average disease score below the empirical group of ‘resistant’ lines and an average disease score above the empirical group of ‘susceptible’ lines was recorded.

## Results

### Phenotypic Variation of LLS Resistance

**FIGURE 1:**
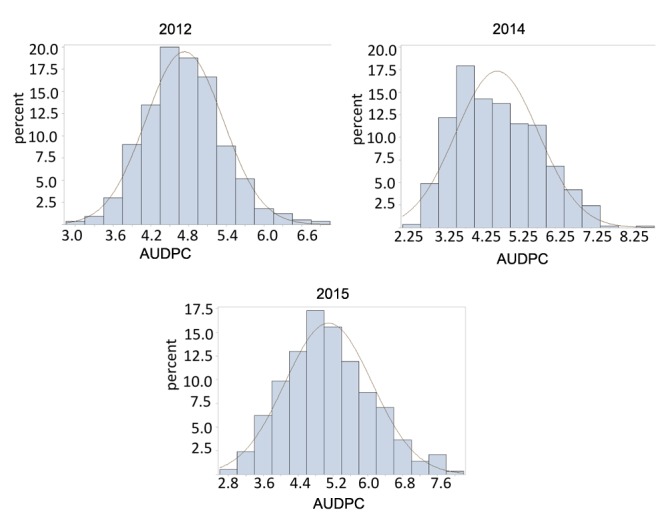
Phenotype distribution of Area under disease progress curve (AUDPC) for late leaf spot (LLS) disease. The normal distribution curve in the graph represented the expected percentage of recombinant inbred line (RILs) with respect to disease score range.

Late leaf spot ratings (AUDPC) were significantly different between Florida-07 and GP-NC WS 16 in 2012 and 2015 but the difference was not significant in 2014 (**Table 1**). The RIL population demonstrated large continuous phenotypic variation across all three years (**Figure 1**) and transgressive segregation for leaf spot resistance was observed (**Table 1**). Both RIL and environment (year) significantly affected LLS resistance in the analysis of variance test whereas the effect of RIL × environment (year) was not significant (**Table 2**). The year to year variation in phenotype data is caused by the fluctuation of disease pressure in the natural field environment; however, the lack of RIL x environment effect indicates that the RILs performed consistently in response to varied levels of disease pressure. The broad sense heritability was 0.88 supporting high genetic influence on the phenotypic variation. Since the RIL data deviated from a normal distribution detected by the Shapiro test, square-root transformation was applied to normalize the data set.

**Table 1:** Area under disease progress curve (AUDPC) of Florida-scale rating for late leaf spot (LLS) disease in the recombinant inbred line (RIL) population and parents.

Environment	Florida-07	GP-NC WS 16	RIL range	*Mean*	*SD*	Skew	Kurt	w (sig.)
Year 2012	4.9	3.8	3.1–6.9	4.7	0.6	0.34	0.46	0.99 (0.003)
Year 2014	3.6	4.2	2.3–8.8	4.6	1.2	0.40	–0.48	0.98 (0.0001)
Year 2015	5.8	3.9	2.8–7.9	5.1	1.0	0.37	–0.07	0.99 (0.0001)

*SD, standard deviation; Skew, Skewness; Kurt, Kurtosis; w, Shapiro–wilk statistic value; sig., significance.*

**Table 2:** Analysis of variance and broad sense heritability for LLS in the RIL population across three environments.

Variables	Mean square	df	*F*-value	*P*-value	*h*^2^
RIL	3.63	192	7.3	<0.001	0.88
Environment	33.61	2	67.4	<0.002	
RIL × Environment	0.48	384	1.0	0.6607	
Error	0.33	1136			

### Identification of LLS Resistant and Susceptible Bulks

Selection of individuals to include in sequencing bulks was done by ranking each line for every year and selecting the top five and bottom five lines according to average rank (**Table 3**) The median bulk ranking for the “Resistant” bulk (R bulk) was 7.75 and the median ranking for the “Susceptible” bulk (S bulk) was 177.5. These bulks were subjected to whole genome shotgun sequencing.

**Table 3:** Ranks of individuals making up ‘Resistant’ and ‘Susceptible’ bulks.

Line	Mean Rank	2012	2013	2014	2015
	**LLS**				
**“Resistant” Bulk**					
1028	4	5	8	1	2
952	5.5	18	1	2	1
1036	7.75	8	11	8	4
980	21.25	15	2	13	55
954	9.25	21	4	7	5
GP-NC WS 16	39.75	8	1	149	1
Bulk median	7.75	15	4	7	4
**“Susceptible” Bulk**					
1012	177.5	171	181	179	179
1042	180.25	180	178	173	190
924	177.25	179	180	176	174
1075	177.25	184	175	184	166
917	188	189	189	182	192
Florida-07	114.5	126	72	102	158
Bulk median	177.5	180	180	179	179

*Ranks for each line are indicated for each year of data considered with the median rank for the bulk for each year at bottom.*

### False Positives Result in Too Much Noise in 4× Peanut

Calling SNPs in peanut, like other polyploids, is difficult because of highly similar homeologous sequences between subgenomes. A hypothesis is that any homeologous false positive SNP can be ignored because they would appear neutral between bulks and would not affect the identification of significant SNPs. An analysis was first carried out using methods similar to those applied to inbred diploid species such as rice, cucumber, or tomato (Takagi et al., 2013; Lu et al., 2014; Illa-Berenguer et al., 2015). As expected, an extraordinary number of putative ‘SNPs’ were called between bulks; 2,245,504 after filtering for depth, indicating a large number of false positive SNP calls. After calculating ΔSNP and using a sliding window smoothing approach, the high number of false-positive SNP calls created too much noise to identify significant regions (Supplementary Figure S1). A different approach was needed to analyze these data appropriately.

### SWEEP Filtering Identifies Significant QTL

**FIGURE 2:**
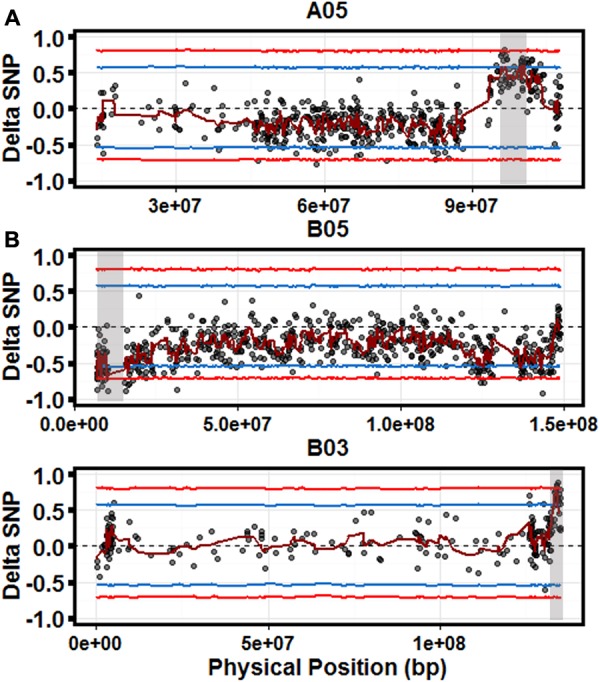
Quantitative trait loci (QTL)-seq identifies significant QTL for controlling LLS resistance. Scatter plots for chromosomes A05 (top), B05 (middle), and B03 (bottom). Each graph is a scatter plot of each ΔSNP (R-Bulk SNP Index–S-Bulk SNP Index) plotted against the physical position based on the *A. duranensis* **(A)** and *A. ipaensis* **(B)** pseudomolecules. The dark red line represents a sliding window of 2 Mb moving 500 kb intervals. Statistical confidence intervals under the null hypothesis of no QTL are plotted for each marker (blue – *p* < 0.05 and red – *p* < 0.01). Gray shaded boxes indicate significant QTL.

A total of 5,513 parental SNPs (2,489 A; 3,024 B) were detected in the bulks, using pipeline specifically designed to deal with the particular issues in polyploid genomes, SWEEP (Clevenger and Ozias-Akins, 2015). Significant candidate QTLs were identified on three chromosomes, A05, B03, and B05 (**Figure 2**) with most significant sliding window ΔSNP values of 0.6, 0.78, and –0.74, respectively (*p* < 0.05). The estimated regions for each QTL are 4.7 Mb (A05), 1.2 Mb (B03), and 3.4 Mb (B05). The candidate QTL on chromosomes A05 and B03 represented alleles from the resistant parent (GP-NC WS 16) as expected. The QTL on B05 represented alleles from the susceptible parent Florida-07. Three Kompetitive Allele Specific PCR (KASP) markers were developed representing significant SNPs within each QTL region (Supplementary Table S1). These markers were used to genotype the population and retroactively select for putative resistance and susceptibility alleles. Linear regression of each marker over the 4 years of data revealed the markers explained 10, 5, and 2% of the variance (B05, B03, and A05, respectively). All three markers explained 15% of the observed variance.

### Validation of Identified Regions

**FIGURE 3:**
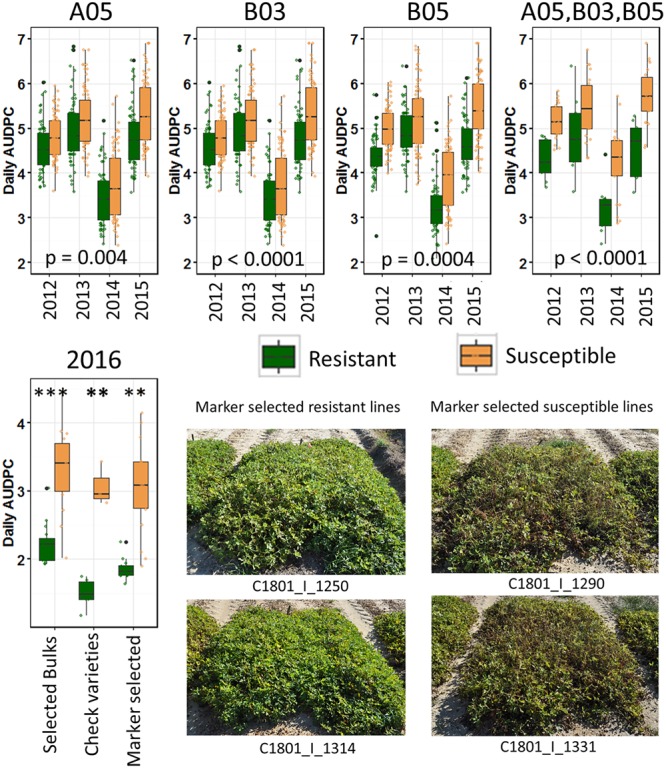
Validation of identified resistance QTL. (Top) Lines with putative ‘resistant’ alleles and ‘susceptible’ alleles were selected in the RIL population and tested against each other across years with a Kruskal Wallis test. From left to right selected only with marker on A05, B03, or B05, and with all three markers (Top right). (Bottom) Validation test in 2016. Lines selected for bulks, resistant and susceptible check varieties, and lines blindly selected with the three identified markers were grown in an unsprayed test in a completely randomized design with three replicates each of two-row/1.524 m plots. Within each category, asterisks indicate significance by a Kruskal–Wallis test (^∗∗∗^*p* < 0.001; ^∗∗^*p* < 0.01). (Bottom right) Plots of two lines selected for resistance alleles and two lines selected for susceptibility alleles with three markers.

To validate the identified regions, the three KASP markers with ΔSNP values for each SNP of 0.6 for A05, 0.78 for B03, and –0.8 for B05, were first used to genotype the entire population (Supplementary Table S1). Then lines were selected for combined putative ‘resistant’ and ‘susceptible’ alleles with all three markers. The null hypothesis of no effect of the marker on resistance to LLS in the field, tested with a Kruskal Wallis test, revealed significant effect of each marker on leaf spot field resistance across all 4 years of data (**Figure 3**, top). As a confirmation of these results, the data were subjected to a bootstrapping algorithm. After 100 simulations across all 4 years of data, more than 95% of randomly selected pools of individuals had an average disease score greater than the ‘resistant’ group selected with the three markers (*p* < 0.05). Additionally, more than 95% of randomly selected pools of individuals had an average disease score less than the ‘susceptible’ group selected with the three markers (*p* < 0.05).

A blind validation test was conducted to further validate the identified markers. First, lines were selected among the RILs advanced in North Carolina that were not tested for LLS severity previously. The lines were selected only with the KASP markers to select for ‘resistant’ and ‘susceptible’ alleles for each marker. The selected lines were tested in an unsprayed field test with three replicates following the randomized block design. In addition, the lines used in each bulk for sequencing and resistant and susceptible checks were included in the test (**Figure 3**, bottom). The selected lines were significantly different in response to LLS.

## Discussion

QTL-seq is a powerful tool to rapidly identify and deploy markers linked to traits of interest. The method, developed by Takagi et al. (2013), is an extension of bulk segregant analysis (Giovannoni et al., 1991; Michelmore et al., 1991). In contrast to bulk segregant analysis, which was applied to F_2_ individuals to saturate genetic maps in regions of interest with PCR-based markers, QTL-seq utilizes high-throughput sequencing for access to all polymorphisms available. In addition, QTL-seq identifies new regions of interest and depending on the population used, can fine-map the candidate region in a single step. QTL-seq has been used effectively in many diploid crops to identify regions controlling traits of interest, including rice, cucumber, tomato, and chickpea (Takagi et al., 2013; Lu et al., 2014; Illa-Berenguer et al., 2015; Singh et al., 2016). Recently, QTL-seq was used effectively in combination with differential expression analysis to identify candidate genes related to response to boron deficiency in allotetraploid *Brassica napus* (Hua et al., 2016). Mapping-by-sequencing was used to map an early flowering mutant and yellow rust resistance in wheat (Gardiner et al., 2014, 2016), showing that this method can be useful in large polyploid genomes. The current study extends these findings, by showing that by using robust parental SNPs, more than one smaller effect QTL can be identified and deployed immediately using marker-assisted selection even in polyploid crops with large, complex genomes.

For cultivated peanut, the rate-limiting step for performing QTL-seq analysis is having access to high-quality polymorphisms. New pipelines such as SWEEP, developed specifically for allopolyploids, allow more precision for the analysis of QTL-seq data. Without knowledge of robust polymorphisms between parental genotypes in this study, the collective noise of the false positive SNP calls did not allow identification of significant loci (Supplementary Figure S1). After identifying polymorphisms in parents using SWEEP, analysis of those SNPs elucidated three clear candidate QTLs which were then validated in a follow up experiment to provide an increase in field resistance to LLS (**Figures 2, 3**). There is an exception to this and that is when the trait of interest is localized within an alien introgression segment that was introduced using interspecific hybridization methods. In this case, when a segment from a foreign genome is controlling the trait of interest, it is routine to identify these introgressions easily (Clevenger et al., 2017). Given a population that is segregating for this introgression segment and the trait such as in Pandey et al. (2016), the underlying QTL identified is the alien introgression segment itself and so is easily identified. In peanut, and possibly other allopolyploids, in the absence of an alien genome segment, high quality SNPs cannot be detected between two bulked sequences. This is simply because the allele frequencies between polymorphisms that do not control the trait of interest are not known and will be centered around 0.5 with some level of variance associated. The large number of homeologous polymorphisms (between subgenomes) will also appear as neutral polymorphisms with a level of variance. In cultivated peanut, there are more polymorphisms between subgenomes than between genotypes and so the true signal will be drowned out by the false signal. In the situation with an alien introgression, however, all the polymorphisms between the alien genome and the cultivate genome will segregate almost completely allowing for a strong signal to be detected.

For a quantitative trait, such as LLS resistance, bulking individuals based on a single year of data would give spurious results. For example, in this study, bulking after 2012 would give much different results than using any of the other years. Bulking using just the data in 2015 would have yielded the same results, but knowledge *a priori* of the best year is not possible. So, QTL-seq for quantitative traits still requires multiple experiments. However, in QTL mapping the entire population needs to be genotyped with genome-wide markers. This expense increases as the size of the population increases. For example, in the C1801 population, 191 lines needed to be genotyped with a SNP array. If this array costs (for example) $50 per sample, then the genotyping cost is $9,550 plus labor and reagent costs for DNA extraction. Using QTL-seq, only two samples need to be sequenced. The genome size and genome complexity drives the sequence coverage needed, but as an example for peanut, sufficient data could be generated by sequencing each bulk to 10× coverage (used in this study). Using the Illumina coverage calculator^3^ two bulks could be sequenced on one NEXTSeq high output run or over two lanes of HiSeq 2500 high output (with 2× extra coverage). The cost of the library construction ($30 per library) and sequencing (about $5,000) will be less than genotyping the entire population. More cost savings can be recovered by combining many bulks that target multiple traits of interest. Further, because sequencing gives access to genotype-specific markers, the marker most tightly linked to the QTL can be identified and converted to a marker for marker-assisted selection. Using sets of SSR markers or a SNP array designed with markers of more broad applicability, there is less chance to identify markers as strongly linked to the trait.

### Statistical Considerations

Magwene et al. (2011) proposed statistical models for the analysis of QTL-seq data in yeast using the G-statistic. In plants, Takagi et al. (2013) used ΔSNP and a permutation test to derive a null model to define significant candidate QTL regions. The latter is the method of choice among plant QTL-seq studies and has been shown to work effectively. Delta G (G statistic normalized by a smoothing function) values were calculated using the data presented in this study. The top 0.1% of Delta G values were from SNPs located within the identified and validated QTL (Supplementary Table S2) highlighting the congruity of the two methods. One drawback of the G statistic is its inability to provide information on which parental allele is contributing to the trait. Using ΔSNP the researcher can easily tell which parent is contributing the beneficial allele.

**FIGURE 4:**
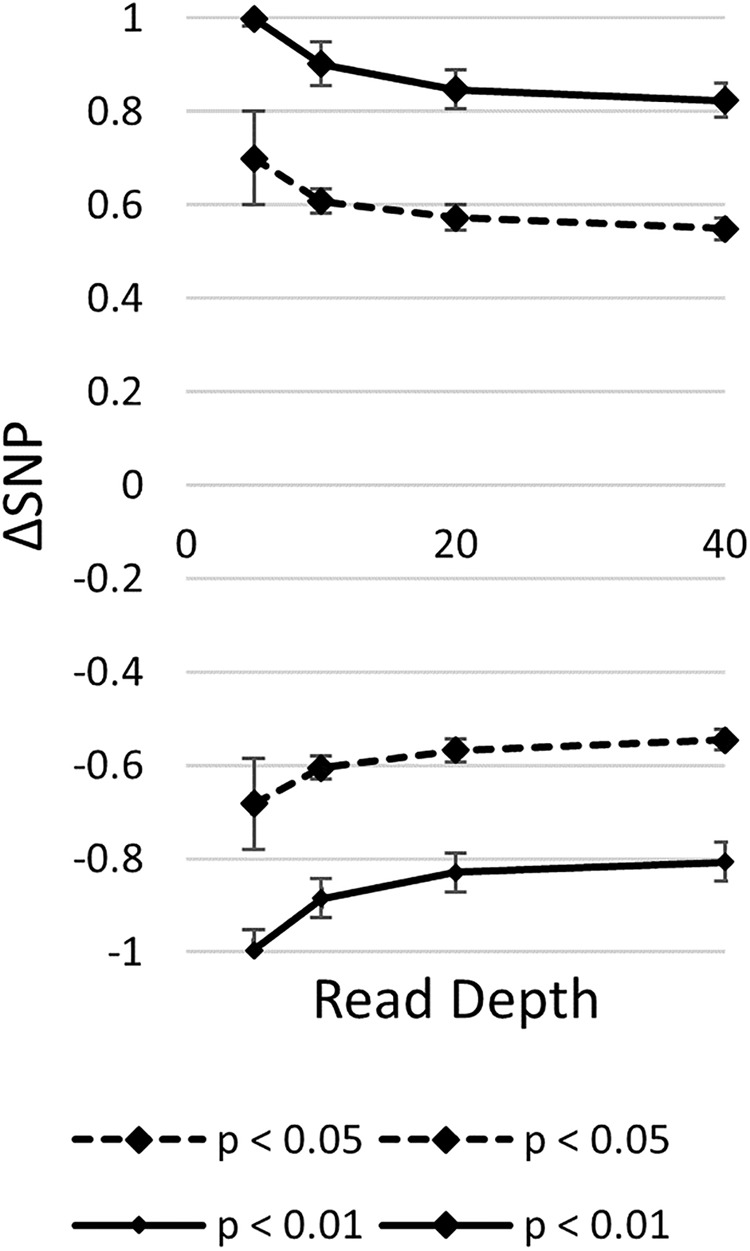
Read depth affects null distribution estimation. A null distribution was generated for each read depth by taking the average and standard deviation of 1,000 runs of the top and bottom 5% (*p* < 0.05) and 1% (*p* < 0.01) of 1,000 simulations for each run.

It is important to note that the probability of the null distribution will change according to the specific read depth at each SNP position. By simulating mapping-by-sequencing datasets, the number of candidate mutations decreased according to increased read coverage and number of pooled individuals (James et al., 2013). Additionally, it was observed that random sampling affected the ability to accurately estimate marker-wise allele frequencies in bulks (Galvão et al., 2012). Random sampling effects increase as read coverage decreases. Although Takagi et al. (2013) generated their permutation test with depth in mind, the null distribution is dataset specific and should be generated for each experiment. To illustrate this, null distributions were simulated for different read depths assuming a biparental RIL population (**Figure 4**). The thresholds for significance varied across read depths. Read depth will vary greatly between different experiments based on experimental design. Variation also is due to sequencer sampling. Random sampling of sequenced reads aligning to each marker, a unique estimation of the null distribution should be established for each marker in a dataset-specific manner. This will control for experiments with high variability of sequencing depth between markers.

## Conclusion

In this study, QTL-seq was used to identify multiple QTLs for LLS resistance in peanut. These QTLs were validated by QTL mapping, backward selection, and blind selection. Markers were designed using the most significant SNPs and it was shown that selection with these markers alone could lead to a significant increase in resistance in the field. The power of this method is its speed and low cost relative to QTL mapping. Further, markers can be designed straight from identified QTLs that are strongly linked to the trait and can be deployed immediately in breeding programs. One caveat is that these QTsL are background specific, as statistical power must be increased substantially to attain perfect linkage to the functional variation. That being said, QTL-seq provides the resolution necessary to find strong linkage in populations where the donor parents of the identified alleles are in the pedigree. QTL-seq can be used for marker-assisted selection even with highly quantitative traits where multiple experiments are used to identify bulks. In many breeding programs, historical data can be leveraged to advise bulk creation, further increasing the efficiency of marker identification.

## Author Contributions

PO-A, JC, and YC conceptualized the research and revised the manuscript; TI, CH, and CC provided the genetic resources and data; JC, YC, CC, SB, and AC performed the experiments, conducted the data analysis, and curated the data; JC and YC wrote the original draft and were responsible for the data visualization; PO-A administered the project.

## Conflict of Interest Statement

The authors declare that the research was conducted in the absence of any commercial or financial relationships that could be construed as a potential conflict of interest.

## Footnotes

1 www.fao.org

2 https://peanutbase.org/

3 http://support.illumina.com/downloads/sequencing_coverage_calculator.html
