# Supplementary material for: Mapping Late Leaf Spot Resistance in Peanut (Arachis hypogaea) Using QTL-seq Reveals Markers for Marker-Assisted Selection
Source: Front Plant Sci. 2018 Feb 5;9:83. doi: 10.3389/fpls.2018.00083 (PMC5807350; doi:10.3389/fpls.2018.00083)
Supplement: Supplementary file 5 [file Image_1.pdf]

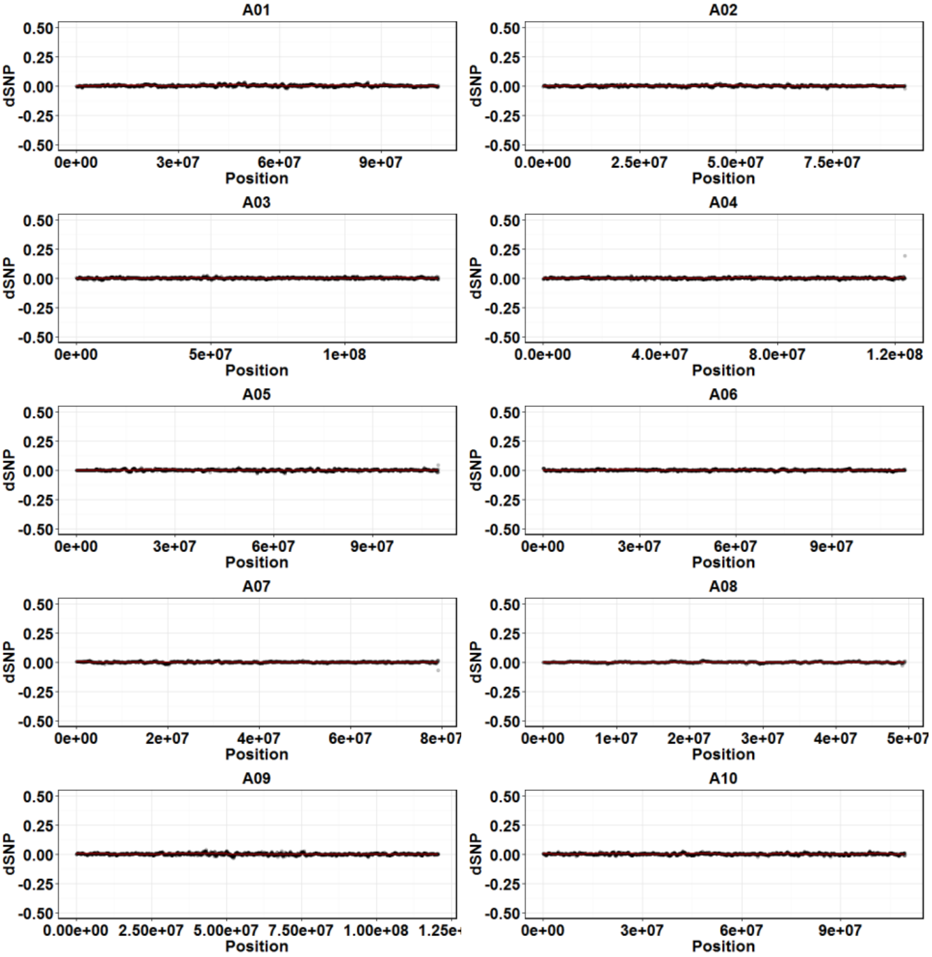
A01
A02
A03
A04
A05
A06
A07
A08
A09
A10
dSNP
Position
0.50
0.25
0.00
-0.25
-0.50
0e+00
3e+07
6e+07
9e+07
0.0e+00
2.5e+07
5.0e+07
7.5e+07
5e+07
1e+08
4.0e+07
8.0e+07
1.2e+08
2e+07
4e+07
8e+07
1e+07
3e+07
4e+07
5e+07
0.00e+00
2.50e+07
5.00e+07
7.50e+07
1.00e+08

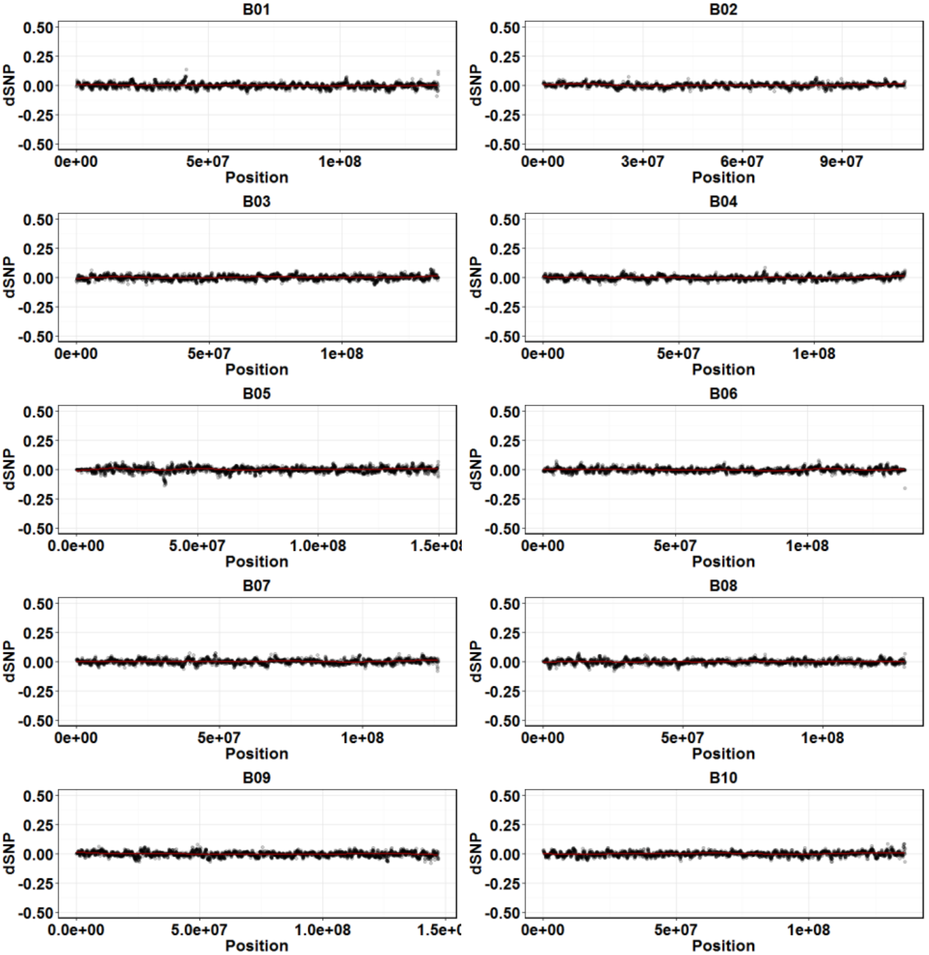

Figure S1 – False positive SNP calls generate noise in analysis. ΔSNP values were generated using all potential SNPs identified in bulks without knowledge of robust parental SNPs. A sliding window of size 2Mb sliding 500kb was used to calculate a sliding average as a smoothing function and plotted for each chromosome. The false positive SNP calls create so much noise that significant regions cannot
